# A Cross-Sectional Study on Anti Hepatitis B Immune Status in Vaccinated Healthcare Workers in the West Pomeranian Region of Poland

**DOI:** 10.5812/hepatmon.850

**Published:** 2012-03-28

**Authors:** Maria Ganczak

**Affiliations:** 1 Department of Public Health, Pomeranian Medical University, Szczecin, Poland

**Keywords:** Medical Staff, Hepatitis B Virus, Vaccination, Testing

## Abstract

**Background:**

Hepatitis B vaccination, recommended for medical staff, has a non-response rate of 5% to 32%. In Poland, there is no standardized postvaccination protocol to verify immunity.

**Objectives:**

To determine the fraction of those who have been vaccinated against HBV (with a complete course followed/not followed by a booster) but not checked for serological evidence of hepatitis B immunity and to detect anti-HBs levels in this group by anonymous cross-sectional sero-survey.

**Patients and Methods:**

Surgical/gynecological staff from 16 randomly selected hospitals in West Pomerania, Poland, were surveyed between July 2010-January 2011. EIA system version 3.0 was used to detect anti-HBs.

**Results:**

Of 488 participants (439 females, median age 42 years) who were previously vaccinated (1-21 years ago), anti-HBs status was not determined after HBV vaccination in 361 individuals (74.0%; 95% CI: 69.9-77.7%), 5% (18/361) of whom had an anti-HBs titer of 0.0 mIU/ml (12/18 who were given booster doses developed anti-HBs > 10 mIU/ml) and 7.2% (26/361) of whom had an anti-HBs titer of 0.1-10 mIU/ml. The multivariate logistic regression model revealed that working in a teaching hospital was associated with lower odds of not being checked for anti-HBs after HBV vaccination (OR 0.22, 95% CI: 0.14-0.35; P = 0.0001).

**Conclusions:**

The lack of a strict post-HBV vaccination policy to confirm immunity results in the majority of surgical/gynecological staff not checking their anti-HBs levels after HBV immunization. It is unknown whether the absence of current serological evidence of hepatitis B immunity can be attributed to non-response, the waning of vaccine-induced immunity, or preserved anamnestic response. The lack of a booster vaccination response in a fraction of subjects suggests that they are non-responders. Strict post-vaccination testing to document immunity remains the key practice to detect non-responders among medical staff.

## 1. Background

Due to the frequent involvement in invasive procedures, surgical and gynecological staff are at a higher risk of acquiring a blood-borne infection through occupational exposure to blood while carrying out duties related to patient care [1]. The risk of occupational hepatitis B virus (HBV) infection is 3-5 times greater than for the general population and increases with age and length of employment [1][2][3]. The sero-prevalence of anti-HBc among surgical staff in Poland varies from 16% among nurses in various surgical and gynecological wards to 40% among those working in orthopedic departments [3][4][5]. A prevalence of anti-HBc of 13-18% has been reported among surgical staff elsewhere [6][7][8]. Because the risk is thought to be higher in relation to the general population [1][3], HBV vaccination is strongly recommended for those working in the health care sector. A routine vaccination policy began in Poland in 1989, with hospitals responsible for the cost of vaccinating employees [3]. The most recent survey of surgical nurses and midwives, conducted in 2009, revealed 100% vaccination coverage [3]. The implementation of a mass immunization program of health care workers (HCWs) has decreased the incidence of HBV dramatically [5][9]. A response to HBV vaccination is determined by the development of hepatitis B virus surface antigen antibodies (anti-HBs), which are detectable in 68-95% of individuals 1-2 months after a complete vaccination schedule [10][11][12]. The response should be controlled in high-risk individuals, such as medical staff, especially surgical staff, because those who are involved in invasive prone procedures may be at risk of not only acquiring infection but also transmitting HBV to patients [1][5]. Those who develop a protective antibody response are protected from infection [1][13]. De Shriver et al. [14] reported an overview of the different policies regarding post-HBV vaccination anti-HBs testing among HCWs from 17 European countries, mainly member-states of the European Union (EU). Poland, with the Czech Republic and Denmark, is in a small group of countries where there are no strict post-vaccination policies to confirm, monitor, or maintain immunity. Various Polish health care authorities have published recommendations stating that "provider units should aim to check the immunity of all HCWs" [15][16]. However, health care facilities may vary with regard to the diligence with which they attempt to comply with the recommendations. In Poland, there is no nationally collected data available to assess the success of the initiative. As a result, we do not know how medical staff follow the recommendations for checking anti-HBs levels after HBV immunization or who the interventions should target to promote it.

## 2. Objectives

The objective of the survey was to determine the percentage of surgical/gynecological staff members who were previously vaccinated but not checked for serological evidence of hepatitis B immunity and to detect anti-HBs levels in this group by an anonymous cross-sectional sero-survey and evaluate the possible effects of the lack of post-vaccination anti-HBs testing on the risk of HBV infection.

## 3. Patients and Methods

Design and Setting: a cross-sectional sero-epidemiological study was conducted from July 2010 to January 2011 of doctors and nurses employed in the surgical/gynecological wards of 16 randomly selected hospitals from the region of Western Pomerania, Poland. Study population and sampling: According to a list of hospitals obtained from the local health department, there are 32 hospitals in the region of Western Pomerania: 12 urban and 20 rural. Multistage, stratified sampling was used for this study, with a random selection of 6 urban hospitals and 10 rural hospitals. In the next step, a stratified sampling of wards proportionate to the numbers of surgical/gynecological wards was selected for each hospital. Unique code numbers were assigned to all subjects to preserve confidentiality. Samples were obtained from all eligible staff who provided written informed consent to participate. Study instrument: A self-administered questionnaire included questions on demographics, including age, gender, and job category; hospital characteristics (type of ward, type of hospital); past history of confirmed HBV immunization (with/without checking for anti-HBs); and receipt of a booster dose. Enzyme immunosorbent assay (EIA) system version 3.0 was used to detect anti-HBs (Abbott Laboratories Inc., Abbott Park, Il, USA). Testing was performed in the referential laboratory of a teaching hospital. Vaccinees were considered seroprotected when their anti-HBs titer was > 10 mIU/ml [12][16]. The antibody titers after vaccination were classified into 4 groups: ≤ 10 mIU/ml (non-responders), 10.1-99 mIU/ml (low responders), 100-999 mIU/ml (medium responders), and ≥ 1000 mIU/ml (high responders) [10]. Statistical analysis: Data were entered and validated using STATISTICA PL Version 7.1. (StatSoft Inc., 2005). Our primary outcome variable was a lack of anti-HBs checking after HBV vaccination, and we aimed to identify staff characteristics that were associated with this outcome. Our secondary outcome variable was anti-HBs level. In the univariate analysis, categorical variable groups were compared by chi-square test with Yates' correction and Fisher's exact test, and Mann-Whitney U test was used for numerical variables. When more than 2 hypotheses were tested, Bonferroni's correction for multiple comparisons was used (adjusted for the number of hypotheses). All significant variables (P < 0.05) in the univariate analyses were entered into the logistic regression model with R software [17]. Age, gender, job category (nurse/midwife/doctor), type of ward (surgical/gynecological), and type of hospital (teaching/ municipal/provincial) were taken into account as potential predictors of not being checked for serological evidence of immunity after HBV vaccination.

## 4. Results

Of the 590 eligible personnel, 488 individuals (82.7%) agreed to participate, 439 (90%) of whom were female. The median age was 42 years (range: 21-60 years). There were 424 nurses (86.7%) and 65 doctors (13.3%)-245 (50.2%) of them worked at surgical wards, 93 (9.1%) at gynecological wards, 109 (22.3%) in operating theaters, and 41 (8.4%) in the admissions area; 282 (57.9%) worked at urban hospitals (39.1% at teaching hospitals, 18.6% at municipal), and 206 (42.1%) at provincial hospitals.

### 4.1. HBV Vaccination

Among 488 subjects who agreed to participate in the study, 22 (4.5%) were vaccinated with only 2 doses of HBV vaccine, and 191 (39.1%) received 3 doses; 275 (56.4%) completed the vaccination schedule and were given a booster dose. All were vaccinated with yeast-derived recombinant vaccine (Engerix B, SmithKline Beecham Biological, Rixensart, Belgium). Of those who were immunized for HBV (time of the last administered dose varied from 1 to 21 years), 485 were able to give a history of their vaccinations: 235 (48.5%) were asked to be vaccinated before starting work, 210 (43.3%) took a course while working at health care facilities, 14 (2.9%) were vaccinated in childhood, 14 (2.9%) were vaccinated before planned surgery, and 12 (2.5%) gave other reasons.

### 4.2. Checking for Anti-HBs After HBV Vaccination

**Figure 1:**
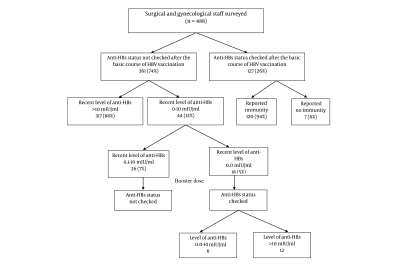
The Scheme of Anti-HBs Status After the Basic Course of HBV Vaccination and the Recent Level of Anti-HBs Among Surgical and Gynecological Staff

In 361 (74.0%; 95% CI: 69.9-77.7%) participants, anti-HBs levels were not checked after the basic course of HBV vaccination (Figure 1). Of the 127 subjects who had titers that were previously checked, 120 (94.5%) reported immunity.

#### 4.2.1. Risk Factors for Not Being Checked for Anti-HBs After HBV Vaccination

No statistically significant differences were observed in the rates of not being checked for anti-HBs after HBV vaccination between gender (P = 0.79), job category (P = 0.44), and type of ward (P = 0.26). The lowest rate of those who were not checked occurred in those < 30 years (23/40; 57.5% were not checked), differing significantly (P = 0.04) compared to the rates in those aged 31-40 years (121/163; 74.2%), 41-50 years (144/188; 76.6%, P = 0.01), and > 50 years (73/97; 75.3%, P = 0.04). In addition, the lowest rate of those who were not checked was observed in university hospitals (107/191; 56.0%), differing significantly (P = 0.0001) versus municipal (77/91; 84.6%) and provincial hospitals (176/206; 85.4%). Variables that were significant at the univariate level were then entered into a stepwise logistic model, which revealed that working in a teaching hospital was associated with smaller odds of not being checked for serological evidence of immunity after HBV vaccination (OR 0.22, 95% CI: 0.14-0.35; P = 0.0001).

###  4.3. Prevalence of Anti-HBs

In those whose anti-HBs status was not determined after the basic course of HBV vaccination, 317/361 (87.2%) subjects (Figure 1) were found to have a recent level of anti-HBs > 10 mIU/ ml; in 44/361 (12.2%) subjects, the recent level of anti-HBs was 0-10 mIU/ml (among them, 18/361 [5.0%] had a recent level of anti-HBs 0.0 mIU/ml). All potential non-responders from the group with the recent level of anti-HBs 0-10 mIU/ml were asked to immunize themselves with a booster dose of HBV vaccine and to check anti-HBs levels 1-2 months later. Although the entire group completed the booster, only participants from the sub-group with an anti-HBs level of 0.0 mIU/ml agreed to check their antibody levels later; 12 of 18 developed anti-HBs > 10 mIU/ml. Of the 26 participants with a recent level of anti-HBs of 0-10 mIU/ml who did not check their antibody level after taking the booster, 22 belonged to a group that was previously vaccinated with only 2 doses of HBV vaccine.

## 5. Discussion

Anti-HBs is an important serological marker for vaccine-induced immunity to HBV. Therefore, the practice of serotesting after HBV vaccination is widespread. Recommendations for post-HBV immunization serology for persons who are at increased risk of infection exist in many countries, such as the US, Canada, Australia, and India [18]. Under EU legislation, employers are responsible for protecting their HCWs against blood-borne infections [19]. The practice of serotesting after HBV vaccination is applied in the vast majority of European countries, not only for doctors and nurses but also paramedical, technical, and cleaning staff [14]. Data from a large tertiary referral teaching hospital in Dublin, Ireland, revealed that 93% of nurses reported that hepatitis B antibody levels were checked on completion of the immunization [20]. In a cross-sectional survey that was conducted in a teaching hospital in Paris, France, among various job categories, the HBV vaccination rate was 93%, with 65% of respondents being aware of their immune status [21]. As discussed, Poland, Denmark, and the Czech Republic are the only respondent countries in the European survey of hepatitis B vaccination polices that do not perform serological testing [14]. The lack of strict post-HBV vaccination policy to confirm immunity has resulted in the majority of Polish surgical/gynecological staff not checking their anti-HBs titers after HBV immunization. Because the situation in Poland is different than in other EU countries, the reasons for not performing serological testing should be considered. Given the current financial condition of health care in Poland, the main issue regarding anti-HBs sero-testing after HBV immunization of HCWs is who is going to pay for it. Although anti-HBs testing is relatively cheap, some health care facilities cannot afford it; thus, the responsibility might fall on the medical staff to cover the cost. However, if the staff covers the cost, they should be informed of the test's real benefits and that it is aimed to simply enhance safety while detecting those who are not aware that they are non-responders. For individuals and policymakers, the balance between health and economic concerns is a critical factor regarding post-HBV vaccination testing. Vaccinated medical staff should be aware of their post-vaccination anti-HBs status, since they may be one of the 5% to 32% of vaccinees who are non-responders and who remain susceptible to HBV infection [10][11][12][15][16]. Our study shows that the majority of surgical/gynecological staff does not routinely check their post-vaccination titers and that 12% of them do not have protective serum anti-HBs concentrations (5% has undetectable titers). It is uncertain whether to attribute the absence of current serological evidence of hepatitis B immunity to nonresponse, waning vaccine-induced immunity, or preserved anamnestic response [22]. The lack of a booster vaccination response in one-third of subjects of those in whom recent anti-HBs levels were 0.0 mIU/ml and who had agreed to check their immunity after a booster dose suggests that they are non-responders. Unfortunately, we were unable to obtain feedback from participants in whom anti-HBs levels, detected recently in our survey, were 0.1-10 mIU/ml and who were asked to immunize themselves with a booster dose of HBV vaccine and check anti-HBs level 1-2 months later (Figure 1). The vast majority of those with anti-HBs levels at 0.1-10 mIU/ml belonged to the group that was vaccinated with only 2 doses of HBV vaccine. It is possible that participants were convinced that the protective effect of a booster dose, even administered years after the second vaccine dose, is identical to that of a third dose taken during a basic course of HBV vaccination. Clearly, their post-vaccination anti-HBs status should still be checked.

The study revealed that working in a teaching hospital is associated with lower odds of not being checked for serological evidence of immunity after HBV vaccination. The possible explanation of this finding is that medical personnel who work at academic hospitals have more opportunities to gain adequate knowledge about the benefits of checking their anti-HBs titers after a basic course of HBV immunization-eg, by participating in various courses, workshops, and conferences on this subject. Although it was not assessed in this survey, better knowledge on infection control issues by those working in teaching hospitals was observed among study participants (Ganczak, unpublished data). The lack of information regarding their initial response to vaccination may render HCWs at risk of HBV infection. Since a small fraction does not exhibit protective anti-HBs titers and because post-vaccination anti-HBs testing is not performed to define non-responders, there may be a substantial population of surgical/gynecological staff who are at risk of contracting HBV infection but are unaware of their serological status. An HBV immunization certificate can offer doctors and nurses a false sense of security, leading some to mistakenly think that HBV vaccination equals protection, and they also may not report occupational exposures to HBV from infected patients. The risk for contracting HBV within a hospital setting remains significant. As illustrated in our previous study, exposure to sharps is common in Polish surgical/gynecological wards: an average surgeon sustains 10 percutaneous injuries per year, and 1 in 2 nurses is exposed to sharps injuries every 12 months [23]. Only 5% of surgical staff use all protective barriers (ie, gloves, masks, eye protection, and gowns) regularly. One in 50 occupational exposures is reported by surgeons versus 1 in 4 by surgical nurses [23]. In addition, the HBsAg prevalence among surgical/gynecological patients is 0.9% [24].

### 5.1. Limitations

Potential limitations of this study include it being confined to HCWs in hospitals from the West Pomeranian region, and it may not be possible to generalize the results for all Polish hospitals. Another limitation was its reliance on self-administered questionnaires, which raises the issue of the reliability of the gathered data. Participation bias is also possible, as the majority of participants was female.

###  5.2. Conclusions and Recommendations

Strict HBV post-vaccination testing to document immunity is critical to detect non-responders among surgical staff who are constantly exposed to blood and remains the crucial means of decreasing the number of HBV-susceptible individuals [14][15][22]. Therefore, it must be incorporated into a vaccination program on a mandatory basis. To reach greater efficiency in post-vaccination anti-HBs testing coverage among Polish HCWs and among other HCWs, greater energy should be spent on lobbying for the creation of relevant legislation acts. Until then, providing doctors who specialize in occupational health with complete information on the risk of hepatitis B and on the benefits of post-HBV vaccination testing to document immunity of HCWs remains an urgent necessity. Prevention program planners should also focus on raising HCW awareness of the issue. However, everyday practice shows that safety warnings could be repeated forever, but what makes the difference is well-enforced regulation. Targeting all groups of HCWs with appropriate education on HBV infection and active involvement of professional groups in the planning and execution of the vaccination program and the practice of sero-testing after HBV vaccination are some of the key points to note for future health campaigns.
